# Supplementary material for: Exercise-Based Interventions Are Effective in the Management of Patients with Thumb Carpometacarpal Osteoarthritis: A Systematic Review and Meta-Analysis of Randomised Controlled Trials
Source: Healthcare (Basel). 2024 Apr 12;12(8):823. doi: 10.3390/healthcare12080823 (PMC11049805; doi:10.3390/healthcare12080823)
Supplement: Supplementary file 1 [file healthcare-12-00823-s001.zip › healthcare-2914866-supplementary.pdf]

Table S1. Search strategy for MEDLINE, EMBASE, CINAHL, PEDro, Science Direct, Cohrane Library	
#1	(carpometacarpal OR carpo- metacarpal OR thumb OR base of thumb OR basal thumb OR basilar thumb OR thumb base OR trapeziometacarpal OR trapezometacarpal OR trapezialmetacarpal OR trapezio- metacarpal OR trapezo-metacarpal OR trapezial-metacarpal OR metacarpophalangeal joint OR hand OR wrist OR finger OR carpal bones OR pollex OR first carpometacarpal OR first carpo metacarpal OR metacarpus OR metacarpal OR carpal OR carpus OR carpo OR carpi OR trapezium OR trapezoid OR trapezial OR CMC)
#2	(degenerative arthritis OR degenerative arthritis OR degenerative arthrosis OR degenerative osteoarthritis OR degenerative osteoarthritis OR degenerative osteoarthrosis OR degenerative osteoarthritis OR arthritis OR arthritis OR arthrosis OR osteoarthritis OR osteoarthritis OR osteoarthrosis OR rhizoarthosis OR rhizarthosis OR osteoarthritis OR arthralgia OR hyperalgesia OR joint diseases OR pain OR acute pain OR chronic pain OR musculoskeletal pain OR breakthrough pain)
#3	(physiotherapy OR physical therapy OR rehabilitation exercise OR generic exercise OR specific exercise OR home exercise OR therapeutic exercise OR exercise program OR training OR training program OR proprioception OR neuromuscular re-education OR dynamic stabilization OR neural mobilization OR exercise therapy OR stretching cast OR splint OR casting OR orthotic devices OR orthosis OR conservative treatment OR conservative treatments OR conservative option OR conservative options OR manual therapy OR manipulation OR mobilization OR strengthening OR joint protection education OR tens OR ultrasound therapy OR electrotherapy OR magnetotherapy OR biofeedback OR massage OR paraffin OR cryotherapy OR hand therapy OR cognitive therapy OR counseling OR self-management OR occupational therapy).
#4	#1 AND #2 AND #3

Table S2. Pain intensity and disability for the use of exercises in addition to a multimodal therapy programme compared with a control group in patients with thumb carpometacarpal osteoarthritis

Outcomes	No. of studies	Comparisons		Effects estimate [95% CI] P-value	Certainty (GRADE)	Heterogeneity
		Average estimate/assumed risk in the exercise group	Average estimate/assumed risk in the control group			
**Mean change in pain rating** Short-term follow-up	2	The mean pain score was 19.4 (range 15 to 21) in 116 participants.	The mean pain score was 37.3 (range 35 to 44) in 114 participants.	**MD**: -21.91 [-36.59, -7.24] P=0.003	⊕[1] **Moderate**	Chi^2: 16.9, (P <0.001) I^2 =94%
**Disability** Short-term follow-up	1	The mean disability score was 26.5 in 86 participants.	The mean disability score was 36.1 in 84 participants.	**MD**:-8.1, [-4.6, −11.5] p= 0.02	⊕[2] **Low**	-

[1] Inconsistency between included studies due to increased statistical heterogeneity
[2] Based on one study with a PEDro score >7
Abbreviations: CI: confidence interval; MD: mean difference

Table S3. Pain intensity and disability for the use of proprioceptive training compared with standard treatment in patients with thumb carpometacarpal osteoarthritis

Outcomes	No. of studies	Comparisons		Effects estimate [95% CI] P-value	Certainty (GRADE)	Heterogeneity
		Average estimate/assumed risk in the proprioceptive training group	Average estimate/assumed risk in the standard treatment group			
**Mean change in pain rating** Very short-term follow-up	5	The mean pain score was 39 (range 25.4 to 57) in 127 participants.	The mean pain score was 46.6 (range 32 to 64) in 203 participants.	**SMD:** -0.76 [-1.30, -0.21] P=0.007	⨁[1, 2, 3] **Very Low**	Chi²:17.34 (P=0.002) I² = 77%
**Mean change in pain rating** Short-term follow-up	6	The mean pain score was 28.6 (range 21 to 52) in 157 participants.	The mean pain score was 36.6 (range 19 to 53.4) in 171 participants.	**SMD**: -0.93 [-1.86, -0.00] P=0.05	⨁[1, 2, 3] **Very Low**	Chi²: 64.89, (P <0.001) I² = 92%
**Mean change in pain rating** Mid-term follow-up	2	The mean pain score was 24.9 (range 19 to 33) in 78 participants.	The mean pain score was 19.6 (range 15 to 29) in 101 participants.	**SMD:** 0.26 [-0.04, 0.55] P=0.09	⨁⨁[2, 3] **Low**	Chi²: 0.67 (P = 0.41) I² = 0%
**Mean change in pain rating** Long-term follow-up	1	The mean pain score was 13 in 36 participants.	The mean pain score was 12 in 48 participants.	**SMD:** -0.05 [-0.38, 0.49] P=0.80	⨁[4] **Very Low**	-
**Disability** Very short-term follow-up	5	The mean disability score was 34.1 (range 23.8 to 60.8) in 127 participants.	The mean disability score was 39.9 (range 24.2 to 62.1) in 125 participants.	**SMD:** -0.94 [-1.68, -0.21] P=0.01	⨁[1, 2, 3] **Very Low**	Chi²:29.2 (P<0.001) I² = 86%
**Disability** Short-term follow-up	6	The mean disability score was 31.9 (range 11.7 to 59) in 148 participants.	The mean disability score was 36.8 (range 27 to 59.8) in 152 participants.	**SMD:** -0.81 [-1.84, 0.23] P= 0.13	⨁[1, 2, 3] **Very Low**	Chi²:79.7 (P=0.01) I² =94%
**Disability** Mid-term follow-up	3	The mean disability score was 27.2 (range 23 to 42) in 80 participants.	The mean disability score was 24.2 (range 20.8 to 31) in 89 participants.	**SMD:** -0.14 [-0.25, 0.52] P=0.49	⨁[1, 2, 3] **Very Low**	Chi²:2.86 (P=0.24) I² =30%
**Disability** Long-term follow-up	1	The mean disability score was 20.4 in 35 participants.	The mean disability score was 20.8 in 48 participants.	**SMD:** -0.03 [-0.47, 0.41] P=0.89	⨁[4] **Very Low**	-

[1] Inconsistency between included studies due to increased statistical heterogeneity
[2] Indirectness of interventions among the included studies
[3] Imprecision results of the included studies
[4] Based on one study with a PEDro score <7
Abbreviations: CI: confidence interval; SMD: standardised mean difference

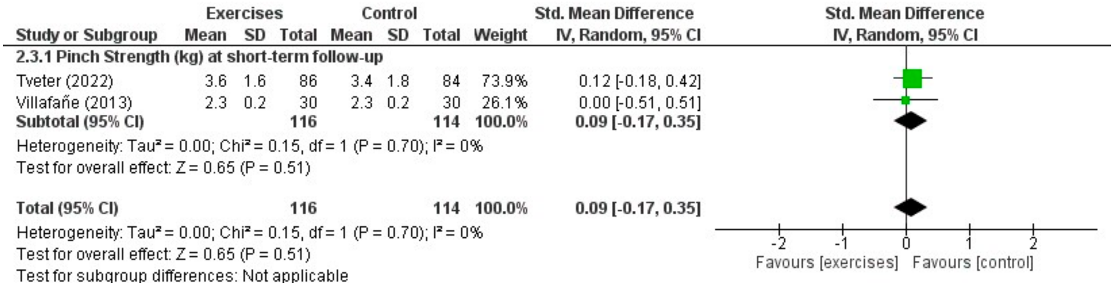

Figure S1. Forest plot for the effectiveness of exercise in addition to a multimodal therapy programme compared with control interventions in pinch strength in patients with carpometacarpal joint arthritis.

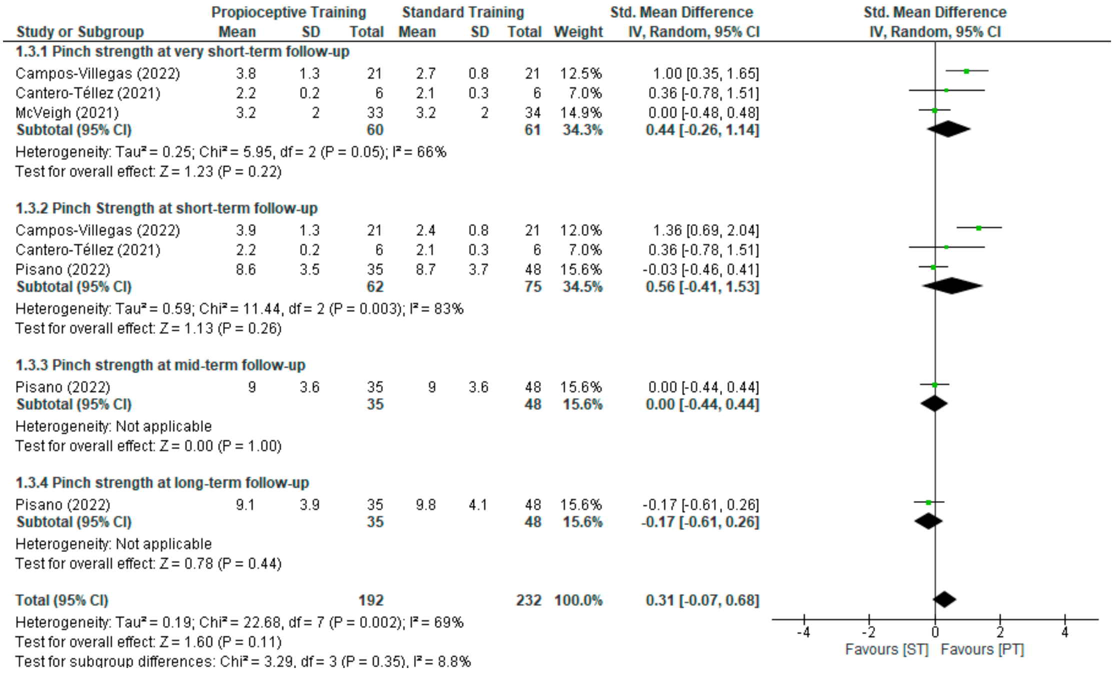

Figure S2. Forest plot for the effectiveness of proprioceptive exercises compared with standard care in pinch strength in patients with carpometacarpal joint arthritis. Abbreviations: ST, Standard treatment; PT, proprioceptive training
